# An Enhanced ELISPOT Assay for Sensitive Detection of Antigen-Specific T Cell Responses to *Borrelia burgdorferi*

**DOI:** 10.3390/cells2030607

**Published:** 2013-09-13

**Authors:** Chenggang Jin, Diana R. Roen, Paul V. Lehmann, Gottfried H. Kellermann

**Affiliations:** 1 Department of Immunology, Pharmasan Labs, Inc., Osceola, WI 54020, USA; E-Mail: diana.roen@pharmasan.com; 2 Cellular Technology Limited, Shaker Heights, OH 44122, USA; E-Mail: paul.lehmann@immunospot.com; 3 NeuroScience, Inc., Osceola, WI 54020, USA; E-Mail: gottfried.kellermann@neurorelief.com

**Keywords:** *Borrelia* infection, T cells, interferon-γ, ELISPOT

## Abstract

Lyme Borreliosis is an infectious disease caused by the spirochete *Borrelia burgdorferi* that is transmitted through the bite of infected ticks. Both B cell-mediated humoral immunity and T cell immunity develop during natural *Borrelia* infection. However, compared with humoral immunity, the T cell response to *Borrelia* infection has not been well elucidated. In this study, a novel T cell-based assay was developed and validated for the sensitive detection of antigen-specific T cell response to *B. burgdorferi*. Using interferon-γ as a biomarker, we developed a new enzyme-linked immunospot method (iSpot Lyme^TM^) to detect *Borrelia* antigen-specific effector/memory T cells that were activated *in vivo* by exposing them to recombinant *Borrelia* antigens *ex vivo*. To test this new method as a potential laboratory diagnostic tool, we performed a clinical study with a cohort of *Borrelia* positive patients and healthy controls. We demonstrated that the iSpot Lyme assay has a significantly higher specificity and sensitivity compared with the Western Blot assay that is currently used as a diagnostic measure. A comprehensive evaluation of the T cell response to *Borrelia* infection should, therefore, provide new insights into the pathogenesis, diagnosis, treatment and monitoring of Lyme disease.

## 1. Introduction

Lyme disease, caused by infection with the spirochete *Borrelia burgdorferi*, is an emerging infectious disease in the United States that has become an important public health problem [1,2,3]. The Centers for Disease Control and Prevention (CDC) reported about 32,500 new cases in 2011 [4], though it is estimated that the actual number might be 10-fold higher, making Lyme disease an epidemic larger than AIDS, West Nile Virus, and Avian Flu combined. Only a fraction of these cases are being diagnosed and treated, due to an unclear history, equivocal manifestations, inaccurate or insensitive laboratory clinical tests, and underreporting [5]. These undiagnosed and untreated patients may develop chronic infection or late stage Lyme disease such as chronic Lyme arthritis [6,7] and chronic Lyme neuroborreliosis [8,9] which can be devastating in some cases. 

The diagnosis of Lyme disease is based primarily on recognizing a characteristic clinical picture [10]. Diagnostic tests for detection of either *B. burgdorferi* itself, or of the ensuing immune response to it have so far been unreliable. Both B cell and T cell immunity develop during a natural infection with *B. burgdorferi* [11,12]. Detection of the specific antibody response against *B. burgdorferi* is utilized conventionally in aiding the clinical diagnosis of Lyme disease. The standard two-tier tests used to detect specific antibodies to *B. burgdorferi* include an enzyme-linked immunosorbent assay (ELISA) and a Western Blot assay (WB) [13]. However, the limitation of these assays is that they have low sensitivity and specificity, frequently producing false negative and false positive results. For example, nearly 30% of results from a Western Blot IgM test are false positive [14]. Furthermore, *Borrelia*-specific antibodies cannot be detected at the early stage of the infection, and a subgroup of Lyme patients lack detectable *Borrelia*-specific antibodies [15,16,17], in both cases providing a false negative result. *Borrelia*-specific T cell immunity has not yet been studied sufficiently due to the lack of highly sensitive and specific T cell-based assays that would be suited for the clinical laboratory. Several attempts have been made to study T cell reactivity against *Borrelia*, but the results were not consistent from different studies [18,19,20]. There is increasing evidence, however, that T cell assays have potential advantages over antibody-based assays in the detection of *Borrelia* infections. Firstly, patients with erythema chronicum migrans (ECM), a clinical manifestation of *B. burgdorferi* infection, displayed specific T cell responses before antibodies to this organism become detectable by ELISA [21,22] and Lastavica *et al.* reported a case in which seroconversion did not occur until 18 months after the onset of the illness [23]. Secondly, a number of patients who received antibiotics for ECM had low or undetectable levels of anti-*Borrelia* antibodies suggesting that the antibody response can be decreased or aborted by early antibiotic intervention [24]. Thirdly, antibody titers often drop to levels below the cutoff value for positivity by ELISA, in particular for untreated subjects or patients with chronic *Borrelia* infection. Fourth, changes in IgM/IgG titers and ratios cannot be used to monitor progress and treatment of *Borrelia* infection since they may stay constant for as long as 20 years [25,26]. Thus, there is a definite need for complementary T cell assays that may help overcome the aforementioned shortcomings of serological assays for diagnosing and monitoring the progress and treatment of *Borrelia* infection. 

The enzyme-linked immunospot assay (ELISPOT) has emerged as a superior method for assessment of the magnitude and the quality of T cell immunity. It enumerates at the single cell level the frequency and cytokine signature of activated antigen-specific T cells [27,28]. The sensitivity of ELISPOT for detecting cytokine producing T cells is 20 to 200 fold higher than that of ELISA or flow cytometry-based intracellular staining [29]. The ELISPOT technology has proven to be extremely sensitive in detecting even low frequencies of antigen reactive T cells and has been approved by the FDA for use in the diagnosis of tuberculosis [30,31]. Here, we explore the potential application of our newly developed Lyme ELISPOT assay, iSpot Lyme, as a diagnostic tool for the detection of Lyme Borreliosis. 

## 2. Materials and Methods 

### 2.1. Isolation of Human Peripheral Blood Mononuclear Cells

Blood donors were either healthy adults without known inflammatory conditions or history of *Borrelia* infection, or subjects with clinically diagnosed Lyme disease. All individuals whom we classified as Lyme patients met the CDC surveillance definition of Lyme disease, including clinical signs and symptoms, history of possible exposure to infected blacklegged ticks, with or without a positive antibody response to *B. burgdorferi* by ELISA and Western Blot, interpreted according to CDC and the Infectious Disease Society of America (IDSA) criteria [32,33]. In addition, non-Lyme control patients with other, specified clinical complications were studied including patients diagnosed with Fibromyalgia, Mononucleosis, Rheumatoid Arthritis and Chronic Fatigue Syndrome. These non-Lyme control patients were from low risk areas of *Borrelia* infection (States ND, MT, UT and AZ) as defined by the CDC. Written informed consent was obtained from all study subjects. Peripheral blood mononuclear cells (PBMC) were separated from acid citrate dextrose (ACD)-treated whole blood using Leucosep tubes (Greiner Bio-One North America, Inc, NC, USA) according to the manufacturer’s instruction. The cell concentration was adjusted to 2.5 × 10^6^ PBMC/mL in CTL Test Plus Medium (Cellular Technology Limited, OH, USA). The cells were kept at room temperature and seeded into the ELISPOT assay 24 h after the blood draw. For the study of inter-assay precision, cryopreserved PBMC from one blood draw were used to avoid biological variation of the test sample. 

### 2.2. ELISPOT Assays with PBMC

All PBMC samples were assayed using the human IFN-γ ImmunoSpot kit by Cellular Technology Limited (OH, USA) per the manufacturer’s instruction. The iSpot Lyme test is made available through Pharmasan Labs, Inc. Briefly, the PBMC were plated into anti-IFN-γ antibody pre-coated 96-well plates at 250,000 cells per well. The PBMC were then stimulated with 10 µg/mL of a proprietary combination of recombinant (r) *Borrelia* antigens purchased from DIARECT AG (Freiberg, Germany). A signal enhancer was added concurrently with the r*Borrelia* antigens and incubated with the PBMC. All culture conditions (negative control, positive control and r*Borrelia* antigen stimulation) were tested in triplicate. The PBMC were incubated for 18–24 h at 37 °C, 9% CO_2_. The resulting ELISPOTs were analyzed using the CTL S6 Ultimate-V Analyzer (CTL, OH, USA) and are reported as IFN-γ Spot Forming Units (SFU). The difference between the iSpot Lyme and the conventional ELISPOT was in the composition of *Borrelia* antigens and in the use of a signal enhancer in the iSpot Lyme assay. The conventional ELISPOT assay followed the identical protocol to the iSpot Lyme assay, but used unenhanced test medium with the r*Borrelia* antigens OspC and VlsE, whereas the iSpot Lyme assay used enhanced medium with a proprietary combination of r*Borrelia* antigens DbpA, OspC, p100, and VlsE.

### 2.3. Measurement of IFN-γ Concentration in PBMC Supernatants

The concentrations of IFN-γ in the supernatant from r*Borrelia* antigen stimulated PBMC were determined using the Bio-Plex suspension array system according to the manufacturer’s instructions (Bio-Rad, Hercules, CA, USA). Briefly, supernatants were collected from 96-well plates containing PBMC that were stimulated overnight with r*Borrelia* antigen, and frozen at −80 °C until use. The thawed supernatant samples were incubated in 96-well filter plates at room temperature for 30 min with antibodies chemically coupled to fluorescent-labeled microbeads. After three washes, premixed detection antibodies were added to each well and incubated for 30 min. Following three washes, premixed streptavidin-phycoerythrin was added to each well and incubated for 10 min. Finally, the beads were washed three times and resuspended with 125 μL of assay buffer. The plates were read on a Bio-Plex 200 reader and data were processed and analyzed by using Bio-Plex Manager Software 6.0 (Bio-Rad, Hercules, CA, USA). Values with coefficient of variation (% CV) above 30 were excluded from the standard curve.

### 2.4. Western Blot Assay

Western Blot assays were performed on patient serum samples by using *Borrelia* Western Blot IgG and IgM kits (Trinity Biotech, Carlsbad, CA, USA) following the manufacturer’s instruction. Briefly, aliquots (20 μL) of undiluted serum samples were added to channels containing the test strips and 2 mL of dilution buffer. Antigens on membranes of this kit were separated by the manufacturer. The IgG kit includes the following 13 bands: p18, p23, p28, p30, p31, p34, p39, p41, p45, p58, p60, p66, and p93; The IgM kit included the following 3 bands: p23, p39, and p14. The strips were scanned using BLOTrix Reader (Frankfurt, Germany). Visualization of specific protein bands indicated the presence of serum IgG or IgM antibodies against *B. burgdorferi-*derived antigens. Samples were classified as positive or negative in accordance with the criteria established by CDC. 

### 2.5. Statistical Analysis

Receiver Operating Characteristic Analysis (ROC) was used to evaluate the accuracy of the tests. The sensitivity was plotted on the y axis, and the false positive rate (1-specificity) was plotted on the X axis. For this purpose, the ELISPOT results of 80 healthy people and 25 Lyme patients were studied. The nonparametric Spearman’s test was used to determine correlations. The nonparametric Mann-Whitney U test was used to compare ELISPOT results between healthy controls, Lyme patients and non-Lyme patients. A *p*-value of < 0.05 was considered statistically significant. The analyses were done by GraphPad Prism 5.0 analysis software (La Jolla, CA, USA).

## 3. Results and Discussion

### 3.1. Enhanced Detection of Borrelia-Specific Reactive T Cells by the iSpot Lyme Assay

**Figure 1:**
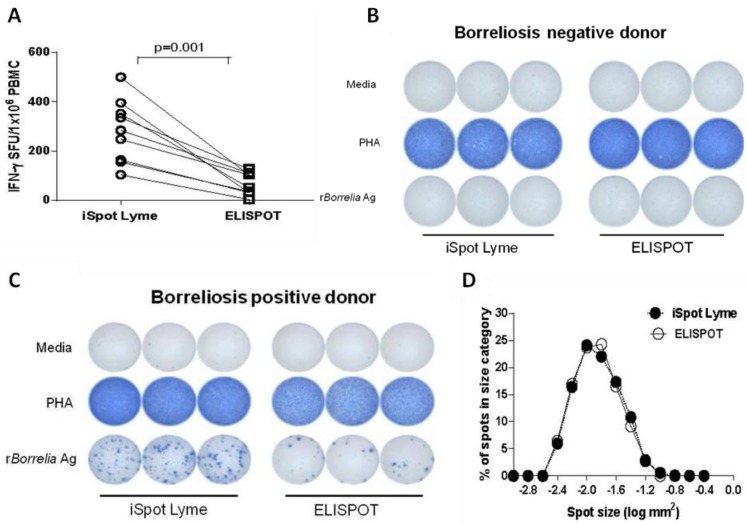
Comparison of detection of *Borrelia*-specific T cells in peripheral blood by the iSpot Lyme assay and conventional ELISPOT assay. (**A**) The frequency of r*Borrelia* antigen-induced IFN-γ spot was established under both conditions in peripheral blood mononuclear cells (PBMC) of *Borrelia* positive patients. Data points obtained from the same donor with the iSpot Lyme assay and conventional ELISPOT assay are connected by a line. Each data point represents the mean spot forming unit (SFU) of triplicate antigen-stimulated wells minus the mean SFU of the corresponding medium control wells. A non-parametric Mann-Whitney U test was used to compare the matched results with a *p*-value of <0.05 considered statistically significant. (**B**) Representative well images for test results obtained from one healthy control run in triplicate and (**C**) from a *Borrelia* positive patient run in triplicate. (**D**) Size distribution of IFN-γ ELISPOTs obtained from the iSpot Lyme assay *vs.* the conventional ELISPOT assay, as specified by the closed and open circles, respectively.

It is well documented that both humoral and cellular immune responses develop in *Borrelia* infection. Assessment of both the function and the frequency of *Borrelia*-specific T cells is crucial for evaluating the cellular immune response to, and diagnosis of *Borrelia* infection [22,34]. Due to the clonal expansion (proliferation) of antigen-specific T cells *in vivo* during an immune response, the presence of increased frequencies of *Borrelia* antigen-specific effector/memory T cells in peripheral blood suggests prior infection/exposure to *Borrelia* [35,36]. To establish the frequencies of *Borrelia*-specific effector/memory T cells in PBMC, we performed ELISPOT assays to measure the numbers of T cells that secreted IFN-γ upon stimulation *ex vivo* by r*Borrelia* antigens. PBMC were isolated from both *Borrelia* positive patients and healthy controls. The cells were plated at 250,000 cells per well and stimulated with recombinant (r) *Borrelia* antigen for 18 to 24 h, followed by the detection of the IFN-γ secreted by the individual T cells resulting in “spots”. The numbers of spot forming units (SFU) were counted by an automated ImmunoSpot reader. To measure antigen-triggered T cell function, we tested the PBMC in a conventional ELISPOT assay and the enhanced Lyme ELISPOT assay (iSpot Lyme assay) in parallel, with a medium that has signal enhancing properties for T cells, CTL Test Plus. Both the conventional ELISPOT and the iSpot Lyme assay were compared for their sensitivity in detection of *Borrelia*-specific effector/memory T cells. The results are summarized in Figure 1. Clearly, the newly developed iSpot Lyme assay significantly increased the sensitivity for detecting *Borrelia*-specific T cells (Figure 1A, *p* = 0.001). More importantly, the iSpot Lyme assay increased the detection of *Borrelia*-specific T cells in *Borrelia* positive samples, without increasing non-specific spots in healthy controls and the medium control background (Figure 1B&C). In addition, the spot size distribution was also analyzed and compared between the conventional ELISPOT and the iSpot Lyme assay permitting us to compare the amount of IFN-γ produced by the T cells under both conditions. As shown in Figure 1D, the spot sizes in the conventional and the enhanced assay showed the normal distribution that is characteristic of the cytokine signature of T cells [37] and there was no size difference between the spots elicited by the two methods. Therefore, the data suggests that our iSpot Lyme assay specifically increases the number of *Borrelia*-reactive T cells that secrete IFN-γ but does not change the IFN-γ productivity of such T cells at the single cell level.

The above results suggest that the iSpot Lyme assay is a highly sensitive *in vitro* assay for the detection of specific T cell immunity to *Borrelia* infection. However, since IFN-γ is secreted by both recently activated T effector cells and resting memory T cells, the iSpot Lyme assay cannot distinguish between active *Borrelia* infection and prior exposure. There is currently no standard laboratory test to distinguish active *Borrelia* infection from prior exposure [38].

### 3.2. Evaluation of the Sensitivity and Specificity of the iSpot Lyme Assay as a Diagnostic Test

**Figure 2:**
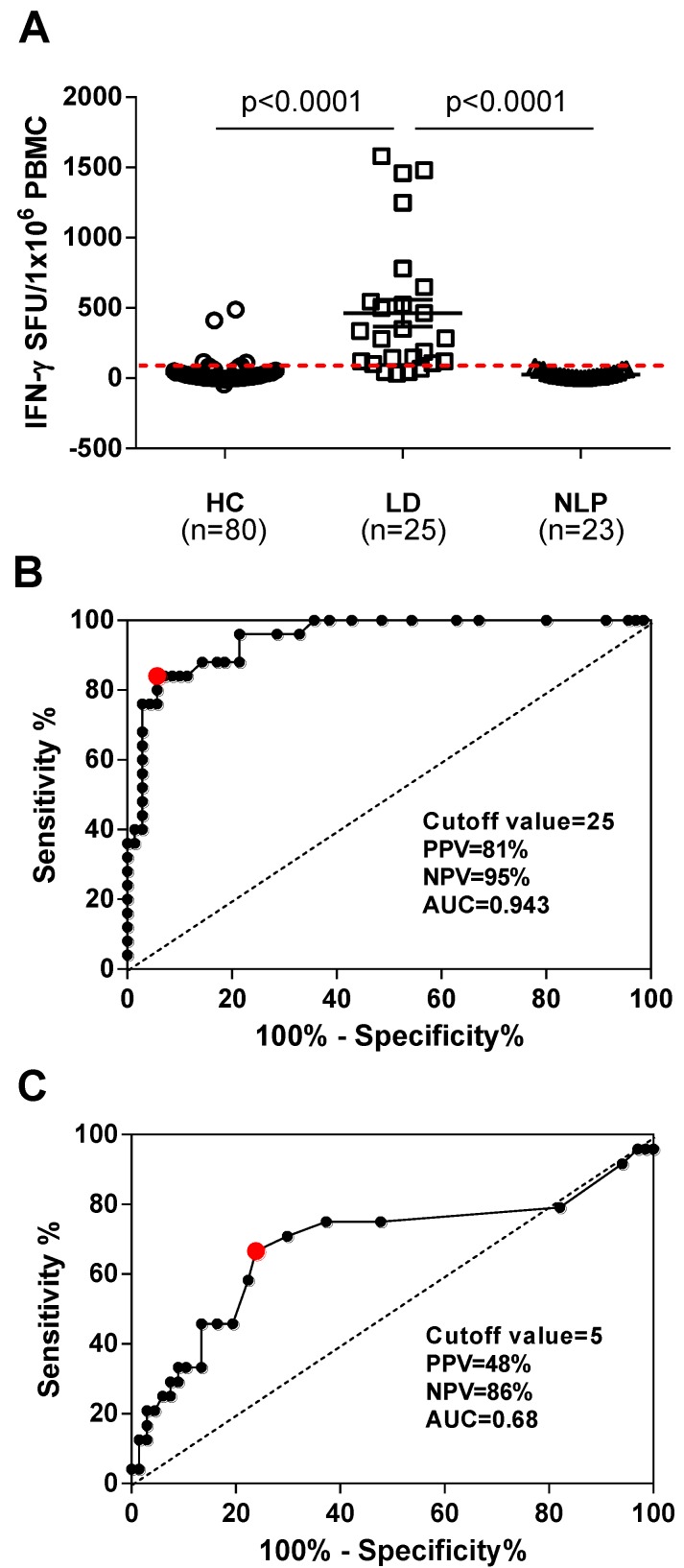
Evaluation of the iSpot Lyme assay as a diagnostic test. (**A**) The results of iSpot Lyme assays performed on 80 healthy controls (HC), 25 clinically diagnosed Lyme disease patients (LD) and 23 non-Lyme patients (NLP) are shown. Each symbol represents the mean SFU obtained in triplicate r*Borrelia*-stimulated wells of a test subject after subtraction of the mean SFU in triplicate medium control wells. Non-parametric Mann-Whitney U test was used to compare the results from LD *vs.* HC and LD *vs.* NLP. A p-value < 0.05 was considered statistically significant. The dotted line represents the cutoff value for positivity at 25 SFU. (**B**) Receiver Operating Characteristics analysis was used to determine the sensitivity, specificity, positive predictive value (PPV), negative predictive value (NPV), area under the curve value (AUC) and cutoff value for the iSpot Lyme assay. (**C**) Receiver Operating Characteristics analysis was used to determine the sensitivity, specificity, PPV, NPV, AUC and cutoff value for the conventional ELISPOT assay.

As the iSpot Lyme assay proved to be a more sensitive tool to detect the *Borrelia*-specific T cells compared with the conventional ELISPOT assay, we next explored if the iSpot Lyme assay could be used as a laboratory T cell-based diagnostic test for *Borrelia* infection. For this purpose, PBMC were isolated from 80 healthy controls that had not been exposed to *Borrelia* (HC), 25 patients with clinically diagnosed Lyme disease (LD) and 23 non-Lyme patients (NLP) who had clinical symptoms similar to Lyme disease. As shown in Figure 2A, the iSpot Lyme assay clearly distinguished the Lyme disease patients from healthy controls and non-Lyme patients, in both cases with a significance level of *p* < 0.0001. To further determine the performance of the iSpot Lyme assay, we analyzed the sensitivity, specificity, the positive predictive value (PPV) and the negative predictive value (NPV) using Receiver Operating Characteristic Analysis (ROC). In this study, the iSpot Lyme assay had a sensitivity of 84% *vs.* 67%, a specificity of 94% *vs.* 76%, a PPV of 81% *vs.* 48%, and a NPV of 95% *vs.* 86% for conventional ELISPOT, respectively (Figure 2B&C). The cutoff value was also determined by ROC as 25 SFU per well for the iSpot Lyme assay. Overall, the ROC analysis suggests that the iSpot Lyme assay fulfills the criteria for a reliable diagnostic laboratory test for *Borrelia* infection with an area under the curve value (AUC) of 0.943 *vs.* 0.68 for the conventional ELISPOT.

### 3.3. Optimization and Validation of the iSpot Lyme Assay

**Figure 3:**
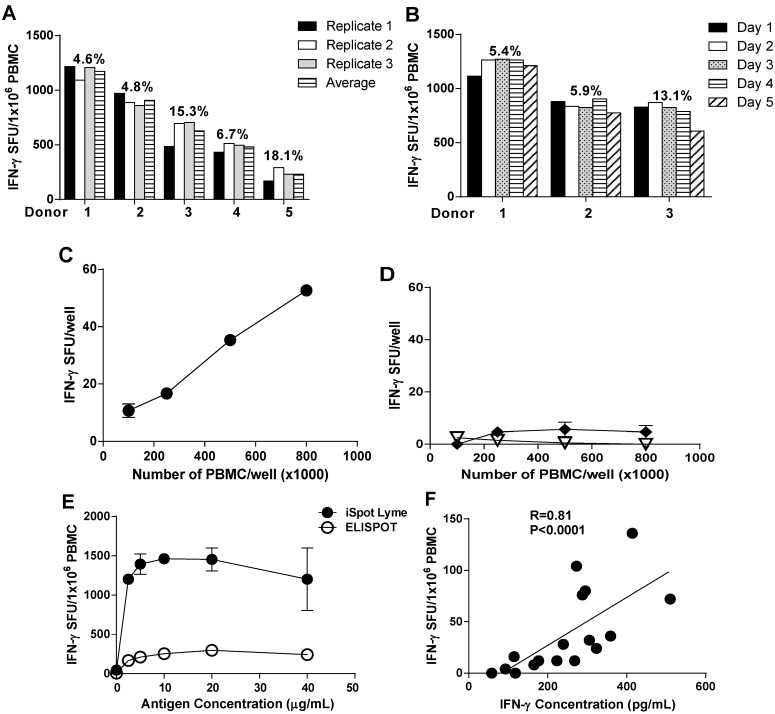
Optimization and validation of the iSpot Lyme assay. (**A**) Intra-assay precision. Five PBMC samples with different r*Borrelia*-triggered SFU response levels were tested in triplicate wells each. Bars with the specified shades show the reactivity for the three individual wells, and the mean of the three. The coefficient of variation for the replicate wells was calculated. (**B**) Inter-assay precision. Cryopreserved PBMC aliquots of the specified three Lyme patients were tested for r*Borrelia* reactivity on five consecutive days. The coefficient of variation for inter assay variation was calculated. (**C**) Relationship between PBMC numbers plated in each well and the IFN-γ SFU in a *Borrelia* positive subject, and (**D**), in a healthy control. Open symbols represent the mean of triplicate antigen-stimulated (treated) wells, the closed symbols represent the mean of the corresponding medium (control) wells. The Standard Deviation (SD) for the triplicate is smaller than the symbol when not visible. (**E**) Dose response curve for r*Borrelia* antigen-stimulated PBMC. (**F**) Correlation of the frequency of IFN-γ secreting *Borrelia*-specific T cells assessed by the iSpot Lyme assay and the concentrations of IFN-γ in the culture supernatant as measured by Bio-Plex suspension array. The nonparametric Spearman’s test was used to determine the correlation. The results showed a *p*-value <0.0001.

To determine the reliability of the iSpot Lyme assay as a routine laboratory test, we performed experiments to study its intra- and inter-assay precision. For the intra-assay precision studies, PBMC from five diagnosed Lyme patients were selected who displayed high, medium and low r*Borrelia*-triggered SFU values. Each of the PBMC samples were run in triplicate. As shown in Figure 3A, the coefficient of variation (CV) among the triplicates ranged from 4.6% to 18.1% with a trend showing that an increase in CV is inversely proportional to SFU values. Inter-assay precision measurements were performed on 3 diagnosed Lyme patient PBMC samples on 5 consecutive days. To make sure that identical cell material was tested, that is, to avoid a biological variation of the sample itself due to blood collections at different times, we used cryopreserved PBMC samples from one blood draw and thawed one aliquot each day for this assay. The CV was 5.4%, 5.9% and 13.1%, respectively (Figure 3B). These data suggested that the iSpot Lyme assay is a reliable test in terms of intra-assay and inter-assay precision. 

The results of an ELISPOT assay can be influenced by the PBMC numbers plated. We therefore tested the relationship between PBMC numbers plated per well, and the r*Borrelia*-induced IFN-γ SFU per well. A linear relationship was seen (Figure 3C), similar to observations made in other antigen-specific ELISPOT systems [39]. The data show that variability in the r*Borrelia*-induced SFU count will depend on the accuracy of cell counting when adjusting the PBMC concentration, and the precision of pipetting. With 250,000 PBMC plated per well, the variability will be directly proportional to the magnitude of such imprecisions. When PBMC of healthy controls were plated in increasing numbers, the IFN-γ spot numbers did not increase with or without r*Borrelia* antigen included in the test system (Figure 3D), suggesting that the IFN-γ spots are produced specifically by *Borrelia*-reactive T cells in response to *ex vivo* restimulation by r*Borrelia* antigen. 

The antigen concentration affects activation of the specific T cells [40]. We therefore tested r*Borrelia* antigen in serial dilution in *Borrelia* positive donors. As shown in Figure 3E, when the antigen concentration was increased, the numbers of SFU also increased initially rapidly but reached a plateau value starting at 10 µg/mL. The r*Borrelia* dose-response curve was very similar for the iSpot Lyme assay and the conventional ELISPOT assay; however, the SFU values were significantly higher in the iSpot Lyme assay. The r*Borrelia* concentration of 10 μg/mL in the iSpot Lyme Test kit, therefore, is safely in the plateau of the dose response curve and inaccuracies in pipetting the antigen are a low risk factor for the test. These results also confirm that the iSpot Lyme assay is superior to the conventional ELISPOT approach for the detection of low frequencies of *Borrelia*-specific effector/memory T cells. 

Assay validation includes the determination of accuracy as established by using an independent readout system for verifying assay results [41]. Therefore, we studied the correlation between IFN-γ SFU numbers as established by iSpot Lyme assay, and the concentration of soluble IFN-γ in the culture supernatants as measured by Bio-Plex method. As shown in Figure 3F, the results of the iSpot Lyme assay were closely correlated to the IFN-γ concentrations as measured by Bio-Plex method (R = 0.81 and *p* < 0.0001). Overall, the validation results showed that the iSpot Lyme assay is a reliable and sensitive test for detecting *Borrelia*-specific T cells with the potential application in clinical laboratory diagnosis for *Borrelia* infection and Lyme disease.

### 3.4. Comparisons between the iSpot Lyme Assay and Lyme Western Blot Assay

**Figure 4:**
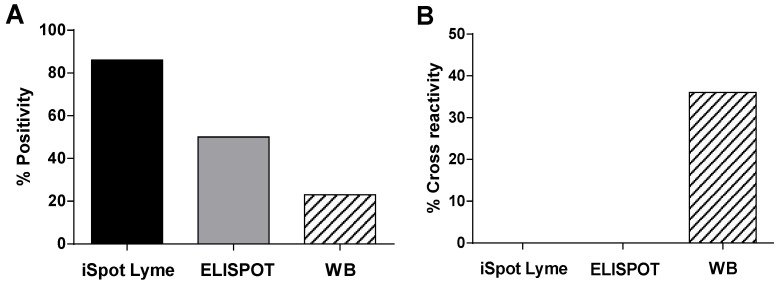
Comparison of sensitivity and specificity for detecting *Borrelia* infection via measuring T cell immunity by ELISPOT *vs.* serum antibodies by Western Blot. (**A**) Lyme ELISPOT assays and Western Blot assay were performed on PBMC and serum of 22 clinically diagnosed *Borrelia* patients. The percentage of individuals who scored positive for each assay is shown. (**B**) Cross-reactivity was assessed in 23 subjects with other clinical conditions, as defined in Materials and Methods, using the iSpot Lyme, conventional ELISPOT and Western Blot assay.

The Western Blot assay has been used conventionally in aiding the clinical diagnosis of Lyme disease [13]. Lyme patients can be classified into two groups according to their serum antibody reactivity to *Borrelia* antigens [24]. Patients whose test positive in Western Blot are seropositive Lyme patients. Accordingly, Lyme patients who do not have detectable antibody levels are defined as seronegative patients. To compare the sensitivity of the iSpot Lyme assay with a Western Blot assay, we performed a study with 23 clinically diagnosed Lyme patients. The Western Blot assay showed 30% positivity in this group of patients whereas the iSpot Lyme assay and conventional ELISPOT showed 84% and 50% positivity, respectively (Figure 4A). In this study, all five patients with positive Western Blot results were also positive for the iSpot Lyme assay. However, in another study we performed, we found that some patients who were positive on their Western Blot assay, had negative results with the iSpot Lyme assay (Data not shown). This discrepancy between the iSpot Lyme assay and the Western Blot assay could be attributed to several factors. One possibility is that there is a dissociation between humoral and cellular immunity to *Borrelia* infection [24]. The other possibility is that the positive results of Western Blot could be false positive as reported by a recent study in which 27.5% of patients who were tested based on suspicion of Lyme disease were found to have a false positive IgM Western Blot result [14]. In addition, we cannot exclude the possibility that T cell responses in some patients may be compromised due to use of immunosuppressive agents or other clinical conditions. 

In summary, these results demonstrate firstly, that the Lyme ELISPOT assay is superior to the Western Blot assay in terms of sensitivity for detecting the underlying *Borrelia* infection. Secondly, the data suggests that there is a dissociation between the magnitude of the humoral and the T cell-mediated cellular immune response in *Borrelia* infection. Thirdly, the data implies that the iSpot Lyme assay may help identify *Borrelia* infected individuals when the serology-based diagnostic fails to do so. 

In addition to the low sensitivity, the Western Blot assay also has relatively low specificity providing frequently false positive results including cross-reactivity with other clinical conditions such as other infectious diseases and autoimmune diseases [14]. To test the cross-reactivity for both the Western Blot assay and the Lyme ELISPOT assay (iSpot Lyme and conventional), 23 non-Lyme patients from low risk areas of *Borrelia* infection were studied. As shown in Figure 4B, the Western Blot assay gave 36% false positive results whereas both the conventional ELISPOT and iSpot Lyme assay did not have any cross-reactivity in the group of subjects studied. Therefore, Lyme ELISPOT assays, in particular the iSpot Lyme assay, are not only more sensitive but also more specific than the standard Western Blot serodiagnostic test for identifying Lyme disease and *Borrelia* infection. Due to the apparent prevalence of either humoral or cellular immunity in infected individuals, it is conceivable that the combination of the iSpot Lyme assay with Western Blot assay would further increase the sensitivity of Lyme disease diagnosis. Studies are ongoing in our laboratory to explore if the iSpot Lyme approach could be used to monitor disease progression and treatment of Lyme disease.

## 4. Conclusions

An enhanced T cell-based immunospot assay for Lyme disease was developed and validated. This iSpot Lyme assay can be used to study the T cell response elicited by *Borrelia* infection, which bridges the gap between the ability to detect humoral immunity and cellular immunity to *Borrelia* infection in Lyme disease. It may be a helpful laboratory diagnostic test for Lyme disease, especially for seronegative Lyme patients. Since serodiagnostic methods of *Borrelia* infection frequently provide false positive results, this T cell-based diagnostic test may help in confirming a Lyme diagnosis. A comprehensive evaluation of both antibody response and T cell response to *Borrelia* infection will provide new insights into the pathogenesis, diagnosis, treatment and monitoring the progress of Lyme disease. 
